# Clinical implications of vasoactive intestinal peptide and 5-hydroxytryptamine in diabetic neurogenic bladder and their relationship with post-void residual

**DOI:** 10.5937/jomb0-62295

**Published:** 2026-02-27

**Authors:** Long Shi, Shaoqi Zhang, Longjun Cai, Jianjun Zhang, Gao Liu, Xiangyu Wang, Furong Ji, Yongming Sun, Zhongqing Wei

**Affiliations:** 1 The Affiliated Suqian Hospital of Xuzhou Medical University, Department of Urinary Surgery, Suqian, Jiangsu, China; 2 The Second Affiliated Hospital of Nanjing Medical University, Department of Urinary Surgery, Nanjing, Jiangsu, China

**Keywords:** vasoactive intestinal peptide, 5-hydroxytryptamine, diabetic neurogenic bladder, post-void residual urine volume, diagnostic and prognostic biomarkers, vazoaktivni intestinalni peptid, 5-hidroksitriptamin, dijabetička neurogena bešika, rezidualni volumen urina nakon mokrenja, dijagnostički i prognostički biomarkeri

## Abstract

**Background:**

To elucidate the clinical significance of vasoactive intestinal peptide (VIP) and 5-hydroxytryptamine (5-HT) in diabetic neurogenic bladder (DNB). We aimed to determine the diagnostic, stratifying, and prognostic value of their simultaneous detection, and to reveal the dynamic associations of these biomarkers with post-void residual urine volume (PVR).

**Methods:**

The study population consisted of 30 DNB patients and 30 healthy individuals from the same timeframe (June 2024-April 2025). Laboratory assessments quantified serum VIP concentration by enzyme-linked immunosorbent assay (ELISA) and 5-HT concentration by liquid chromatography-mass spectrometry (LC-MS). PVR quantification relied on urodynamic examination and abdominal ultrasound. Over a 3-month follow-up, serial measurements of VIP and 5-HT were obtained. Statistical analyses, including Pearson correlation, receiver operating characteristic (ROC) curve analysis, and logistic regression modeling, were employed to assess the relationships of these biomarkers with PVR, bladder function classification, and patient prognosis.

**Results:**

Significant reductions in VIP and 5-HT were observed in DNB patients relative to controls (P&lt; 0.05). The combined use of VIP and 5-HT achieved an area under the curve (AUC) value of 0.878, with 83.33% sensitivity and 86.67% specificity, indicating better diagnostic efficacy than single-marker detection (P&lt; 0.05). VIP and 5-HT levels were inversely related to PVR (P&lt; 0.05). In DNB patients, both biomarkers increased gradually at one and three months post-treatment (P&lt; 0.05), paralleling urodynamic improvements. Stratification by bladder dysfunction severity (mild, moderate, severe) revealed a progressive decline in VIP and 5-HT levels with increasing severity (P &lt; 0 .0 5), confirming their utility in disease staging. Patients with lower VIP and 5-HT levels three months after treatment had a greater propensity for upper urinary tract impairment, urinary tract infections, and disease advancement.

**Conclusions:**

The combined detection of VIP and 5-HT enables effective evaluation of DNB severity, forecasts disease progression, and offers a foundation for timely intervention through dynamic monitoring.

## Abstract (sr)

**Uvod:**

Cilj je bio da se ispita klinički značaj vazoaktivnog intestinalnog peptida (VIP) i 5-hidroksitriptamina (5-HT) kod dijabetičke neurogene besike (DNB). Cilj NAM je bio da utvrdimo dijagnostičku, stratifikujucu i prognostičku vrednost njihovog istovremenog otkrivanja i da otkrjemo dinamičke povezanosti ovih biomarkera sa rezidualnom zapreminom urina nakon mokrenja (PVU).

**Metode:**

Ispitivanu populaciju činilo je 30 pacijenata sa DNB i 30 zdravih osoba iz istog vremenskog perioda (jun 2024 - April 2025). Laboratorijskim analizama kvantifikovana je koncentracija VIP u serumu enzimskim imunosorbentnim testom (ELISA) i koncentracija 5-HT tečnom hromatografijom - masnom spektrometrijom (LC-MS). Kvantifikacija PVR oslanjala se na urodinamski pregled i abdominalni ultrazvuk. Tokom tromesečnog praćenja, dobijena su serijska merenja VIP i 5-HT. Statističke analize, uključujući Pirsonovu korelaciju, analizu ROC krive (receiver operating characteristic) i modeliranje logističke regresije, korišćene su za procenu odnosa ovih biomarkera sa PVR, klasifikacijom funkcije bešike i prognozom pacijenta.

**Rezultati:**

Značajna smanjenja VIP i 5-HT primećena su kod pacijenata sa DNB u odnosu na kontrolnu grupu (P&lt; 0,05). Kombinovana upotreba VIP i 5-HT postigla je vrednost površine ispod krive (AUC) od 0,878, sa osetljivošću od 83,33% i specifičnošću od 86,67%, što ukazuje na bolju dijagnostičku efikasnost nego detekcija jednim markerom (P&lt; 0,05). Nivoi VIP i 5-HT bili su obrnuto proporcionalni PVR (P&lt; 0,05). Kod pacijenata sa DNB, oba biomarkera su se postepeno povećavala jedan i tri meseca nakon lečenja (P &lt; 0 ,0 5 ), paralelno sa urodinamskim poboljšanjima. Stratifikacija prema težini disfunkcije bešike (blaga, umerena, teška) otkrila je progresivan pad nivoa VIP i 5-HT sa povećanjem težine (P&lt; 0,05), što potvrđuje njihovu korisnost u stadijumu bolesti. Pacijenti sa nižim nivoima VIP i 5-HT tri meseca nakon lečenja imali su veću sklonost ka oštećenju gornjih urinarnih traktova, infekcijama urinarnog trakta i napredovanju bolesti.

**Zaključak:**

Kombinovana detekcija VIP-a i 5-HT omogućava efikasnu procenu težine DNB-a, predviđa progresiju bolesti i nudi osnovu za blagovremenu intervenciju kroz dinamičko praćenje.

## Introduction

Diabetic neurogenic bladder (DNB) is a prevalent microvascular complication of diabetes mellitus (DM), affecting approximately 30-50% of patients over the disease course [1]. Central to its pathogenesis is autonomic neuropathy caused by persistent hyperglycemia, affecting both sympathetic and parasympathetic pathways, as well as sacral micturition centers. These changes result in impaired detrusor contractility and incoordination of the urethral sphincter, culminating in clinical manifestations like urinary retention and increased post-void residual urine volume (PVR) [2]. PVR is widely accepted as an important indicator of DNB severity and a predictor of upper urinary tract injury (e.g., hydronephrosis, renal insufficiency) and urinary tract infection [3]. Nevertheless, PVR merely reflects bladder functional status and fails to provide dynamic insights into the underlying pathological progression. Consequently, it has limited utility in the early risk assessment of DNB. Thus, identifying specific biomarkers for early detection, disease stratification, and therapeutic monitoring in DNB remains an urgent priority.

Emerging evidence highlights the connection between neurotransmitters, gastrointestinal hormones, and DNB. Vasoactive intestinal peptide (VIP), a non-adrenergic and non-cholinergic (NANC) neurotransmitter from the enteric and central nervous systems, relaxes bladder smooth muscle and promotes gland secretion to participate in urinary regulation [4]. Research in animal diabetes mellitus models points to a marked downregulation of VIP potentially underlying detrusor contraction dysfunction [5]. The monoamine neurotransmitter 5-hydroxytryptamine (5-HT) is extensively distributed within the central and peripheral nervous systems, where it regulates detrusor contractility and afferent neurotransmission by activating 5-HT receptors [6]. In DNB, clinical evidence links serum 5-HT concentrations to urodynamic findings; yet, its mechanistic role and diagnostic relevance are unclear [7]. Notably, most prior studies have focused on single biomarkers, leaving the combined role of VIP and 5-HT unexplored.

We hypothesize a synergistic interaction between VIP and 5-HT in DNB pathogenesis. This hypothesis is supported by prior evidence of VIP and 5-HT crosstalk in autonomic pathways; for example, animal studies show that VIP potentiates 5-HT-mediated smooth muscle relaxation via shared cAMP signaling [8]. Evaluating both biomarkers simultaneously may yield a composite index reflective of overall bladder neuromuscular function. Confirming their clinical implications in DNB may establish a novel framework for early screening, disease stratification, and personalized treatment strategies, ultimately improving long-term patient outcomes. This exploration addresses an existing research void concerning the combined use of DNB biomarkers while broadening the pathological understanding of DNB from a neuro-endocrine-bladder axis perspective. These findings hold significant promise for clinical translation.

## Materials and methods

### Research design and sample size estimation

In this single-center, cross-sectional observational study, 60 participants (30 DNB patients and 30 DM patients) were targeted for inclusion. Sample size calculation, focused on the correlation between serum VIP/5-HT levels and PVR (primary endpoint), was conducted with G*Power 3.1. Assumptions included a two-sided α=0.05, 80% power, and an effect size of r=0.5 (a moderate correlation between neurotransmitters and PVR based on existing literature) [9]. The computed minimum sample size was 26 per group. Considering a 10% dropout rate, the target enrollment was set at 30 per group.

### Research participants

Two groups - 30 DNB patients and 30 DM patients - were selected during the period from June 2024 to April 2025. DNB group inclusion criteria were: diagnosis consistent with World Health Organization (WHO) DM guidelines [10]; age ≥40 years; disease duration ≥5 years; presence of typical DNB symptoms (e.g., dysuria, urinary retention, frequency, urgency, or overflow incontinence); urodynamic evidence of detrusor underactivity (Pdet_max <20 cm H_2_O) or detrusor-sphincter dyssynergia, with PVR ≥100 mL; and no recent use (within 4 weeks) of agents that alter neurotransmitter metabolism. Control group inclusion criteria included: age- and sex-matched to the DNB group (age ±5 years, 1: 1 sex ratio); no history of neurological disorders, urinary system organic diseases (e.g., benign prostatic hyperplasia, bladder stones), or urinary tract infections; normal urinary ultrasound and urinalysis results, with PVR <50 mL; and no intake of drugs influencing neurotransmitter activity or bladder function in the past 4 weeks. Exclusion criteria for both groups included: the presence of neurogenic bladder attributable to other etiologies; acute urological issues such as infection, calculi, or cancer; severe impairment of cardiac, hepatic, or renal function; pregnancy or lactation.

### Follow-up investigation

DNB patients were managed as prescribed by doctors and followed up for 3 months. DNB patients received standardized treatment including glycemic control (insulin or metformin), α-blockers (e.g., tamsulosin), and bladder training exercises. VIP and 5-HT levels were tracked alongside these interventions. Progression was characterized by either advancement from mild PVR (100-300 mL) to a higher grade (moderate: 300-500 mL; severe: ≥500 mL), or a ≥50% PVR increase in patients with baseline moderate/severe PVR. All enrolled participants completed the study without dropouts, ensuring an intention-to-treat (ITT) analysis. Data from all 30 DNB patients and 30 controls were included in the final analysis.

### Inspection items

Routine examination: Bladder function was assessed by measuring the maximum urinary flow rate (Qmax) with an urodynamic instrument and the PVR via abdominal ultrasound. The PVR measurement was conducted twice immediately after voiding, with the results averaged. Bladder dysfunction was graded as mild (PVR 100-300 mL and Qmax >10 mL/s), moderate (PVR 300-500 mL or Qmax 5-10 mL/s), or severe (PVR >500 mL and Qmax <5 mL/s).

Laboratory examination: Fasting venous blood was collected in both the DNB group (at admission and 1-month and 3-month follow-ups) and the control group (upon admission). An enzyme-linked immunosorbent assay (ELISA) was performed to measure VIP Serial dilutions of VIP standard (0, 10, 20, 40, 80 pg/mL) were added to an antibody-pre-coated 96-well plate for a 2-hour incubation at 37 . Serum was isolated from blood samples via centrifugation. Then, 50 μL of sample serum was added to each well. Meanwhile, blank wells (buffer only) and quality control wells were set up, with incubation carried out at 37 for 1 hour. Post-incubation and three washes with PBST, a biotin-labeled secondary antibody, diluted 1: 1000, was applied, and the plate was incubated for another hour at 37°C. Next, horseradish peroxidase-labeled streptavidin (1: 2000) was added, with incubation proceeding for 30 minutes at 37°C. Color development was initiated by adding TMB substrate for 15 minutes and halted by sulfuric acid. Absorbance at 450 nm was measured, and sample VIP levels were calculated using the standard curve. 5-HT was detected by liquid chromatography-mass spectrometry (LC-MS). Following the addition of methanol (1: 4, v/v) for protein precipitation, serum samples were vortexed for 1 minute and centrifuged (13,000 X g, 10 minutes). The resulting supernatant was isolated and evaporated to dryness using nitrogen gas. The dried extract was then redissolved in 50 μL of a mobile phase (acetonitrile containing 0.1% formic acid: water = 20: 80). Chromatography was conducted with a C18 column (2.1 X 100 mm, 1.8 μm) with gradient elution at 0.3 mL/min mobile phase flow rate. Detection involved electrospray ionization in positive mode (ESI + ), tracking the characteristic fragmentations: 5-HT (m/z 177.1 160.1) and d4-5-HT (m/z 181.1 164.1). Serum 5-HT concentrations were calculated using the internal standard method. Quality control confirmed intra-assay CV <8% and inter-assay CV <12% for ELISA (VIP), and intra-assay CV <5% and inter-assay CV <10% for LC-MS (5-HT), ensuring reproducibility.

VIP was quantified by ELISA due to its high specificity for peptide hormones and compatibility with serum samples. 5-HT was measured by LC-MS for its superior sensitivity in detecting low-abundance neurotransmitters and ability to distinguish 5-HT from metabolic intermediates.

### Ethical statement

All subjects signed a written informed consent form, and the study received approval from the Hospital Ethics Committee. The collection, storage, and detection of biological samples were processed by dedicated technicians to avoid human-induced inaccuracies. This prospective study employed blinding during data collection: VIP and 5-HT examiners were unaware of group assignments, and urodynamic assessments were performed by independent clinicians blinded to biomarker results.

### Statistical analysis

SPSS version 30.0 software (IBM Corp., Armonk, NY, USA) was used for statistical analysis. The comparison of counting data [n(%)] adopted the χ^2^ test or Fisher's exact test (when cell counts <5). Measurement data, confirmed by the Shapiro-Wilk test to conform to a normal distribution, are recorded as (χ±s). Independent samples and paired t-tests were used for comparisons between and within groups, respectively. For longitudinal comparisons of VIP and 5-HT across time points, Bonferroni correction was applied to adjust P-values for multiple testing (significance threshold set at P<0.017 for three time points). Diagnostic performance was evaluated via receiver operating characteristic (ROC) curve analysis. A joint detection model was established according to Logistic regression, and the diagnostic effect was determined based on the maximum Youden index. Pearson correlation was applied for normally distributed bivariate data. A P-value below 0.05 indicated statistical significance.

## Results

### Analysis of inter-group comparability

To establish group comparability, baseline clinical characteristics - including age, gender, family history, and body mass index (BMI) - were compared. No significant differences were observed (P>0.05), and all standardized mean differences (SMD) remained under the 0.4 threshold, confirming that the influence of confounding factors was small. Of note, FPG and PVR were higher in the DNB group than in the control group (P<0.001, Table 1).

**Table 1:** Clinical data sheet of the study subjects.

Projects	Control (n=30)	DNB (n=30)	t or χ^2^ values	P values	SMD
Age	57.67±11.34	61.87±10.51	1.488	0.142	0.380
Gender			0.318	0.573	0.130
Male/Female	20/10	22/8			
BMI (kg/m^2^)	24.00±2.01	24.55±2.38	0.962	0.340	0.250
Family history			0.417	0.519	0.150
Yes/No	5/25	7/23			
Smoking			0.617	0.432	0.200
Yes/No	16/14	19/11			
Drinking			1.071	0.301	0.260
Yes/No	12/18	16/14			
FPG (mmol/L)	10.41±1.78	15.67±4.40	6.076	<0.001	-
PVR (mL)	55.11±22.06	385.68±173.07	10.382	<0.001	-

### Diagnostic utility of VIP and 5-HT in DNB

**Figure 1:**
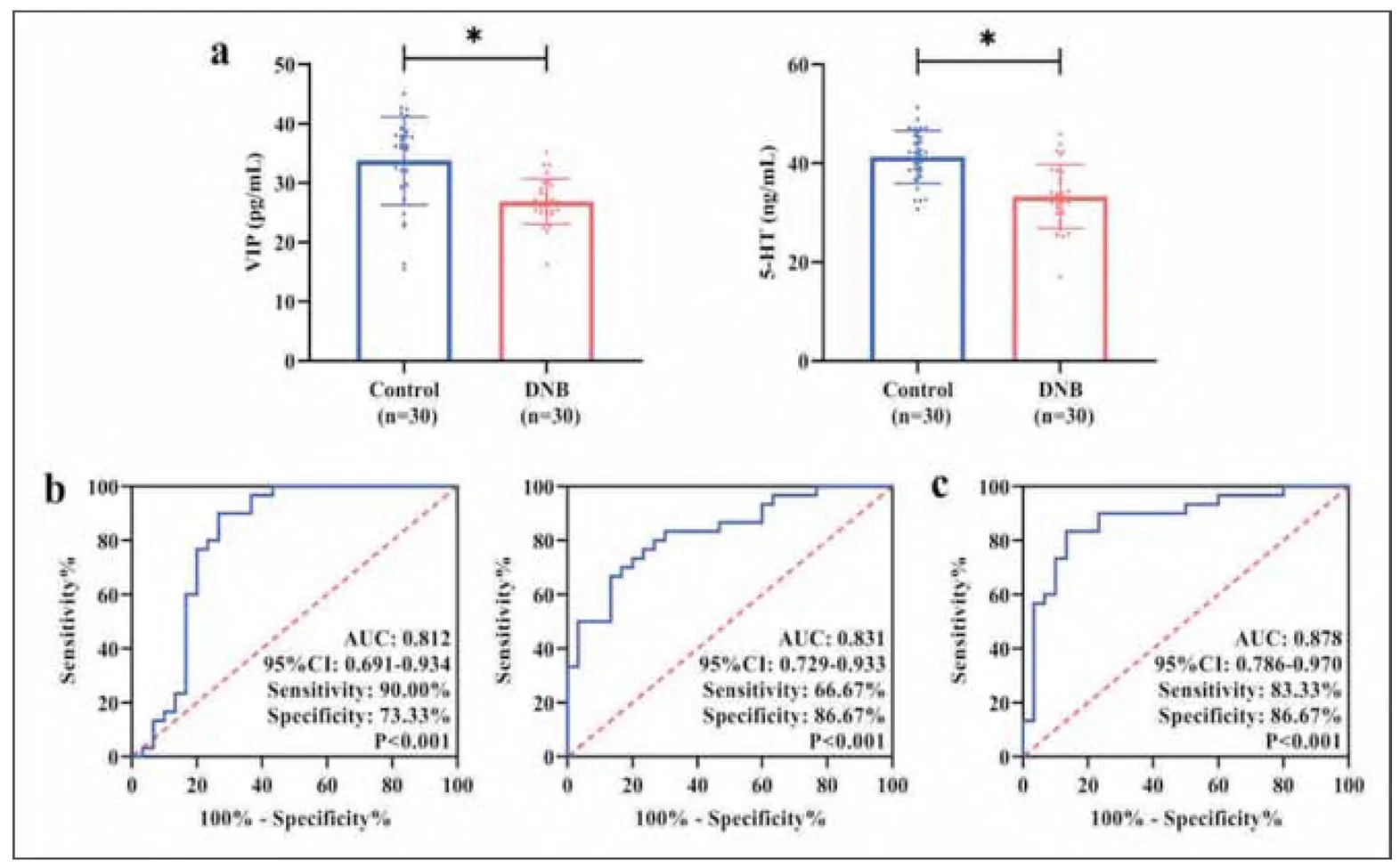
Analysis of the diagnostic efficacy of VIP,5-HT for DNB. (a) Comparison of VIP and 5-HT between DNB and control groups. (b) Diagnostic efficacy of VIP and 5-HT for DNB. (c) The diagnostic effect of combined detection of VIP and 5-HT on DNB. *P<0.05.

Compared with controls, both VIP and 5-HT decreased in DNB patients (P<0.05). ROC curve analysis showed that both markers had certain diagnostic effects on the occurrence of DNB (AUC=0.812, 0.831, respectively). Subsequent regression analysis identified both VIP and 5-HT as independent factors influencing DNB. A combined detection model was constructed based on their regression coefficients (β). Evaluation via ROC analysis revealed an AUC of 0.878 for the combined model (83.33% sensitivity, 86.67% specificity) in detecting DNB, outperforming individual tests (P<0.05, Figure 1).

### Relationship between VIP/5-HT and DNB severity

**Figure 2:**
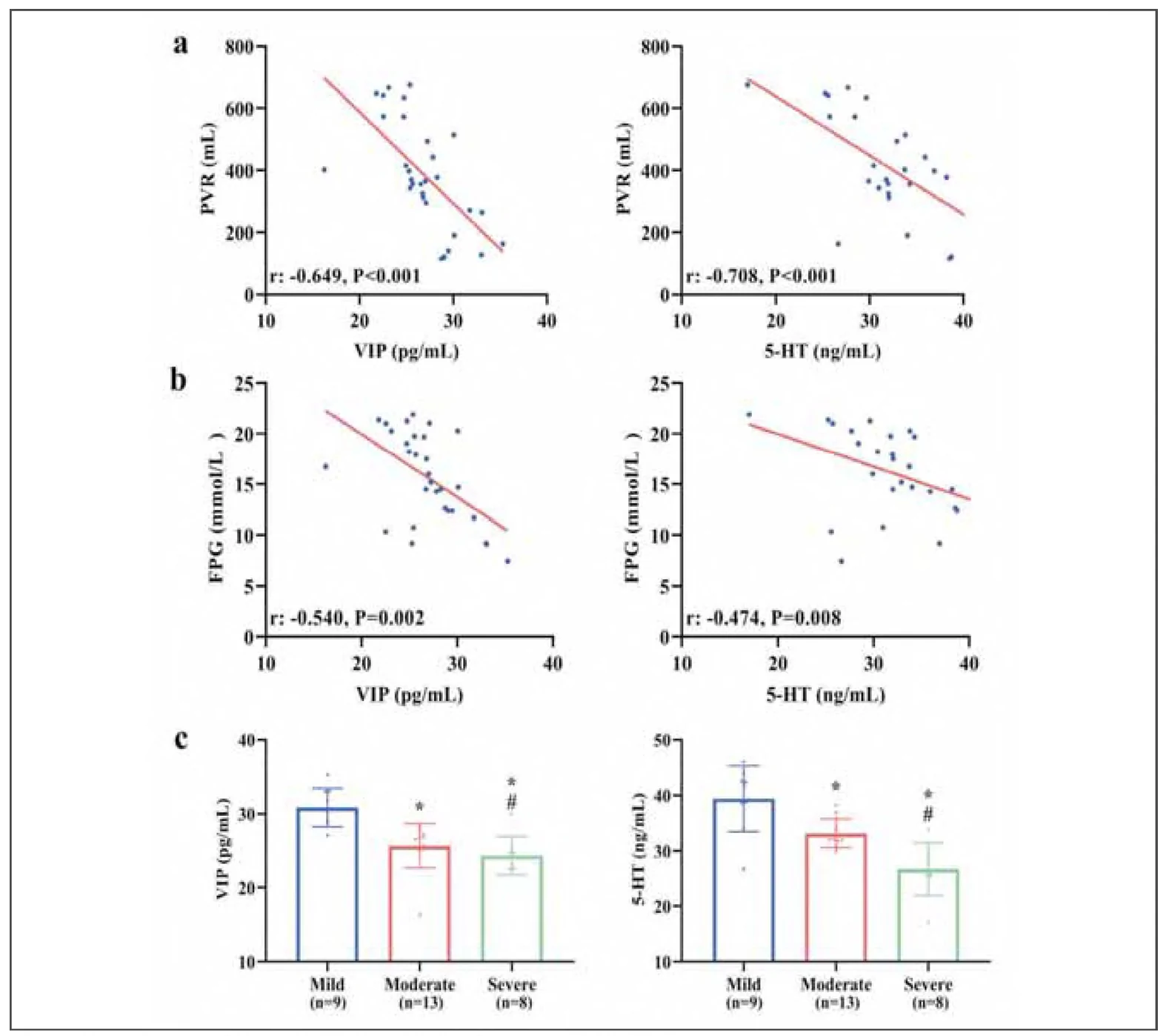
Relationship between VIP,5-HT and disease progression in DNB. (a) Correlation between VIP 5-HT and PVR. (b) Correlation between VIP 5-HT and FPG. (c) Relationship between VIP 5-HT and severity of DNB. * vs. Mild P<0.05, # vs. moderate P<0.05.

According to Pearson correlation analysis, VIP and 5-HT exhibited a significant negative correlation with PVR and FPG among DNB patients (P<0.05). That is, diminished VIP and 5-HT levels correspond to higher PVR and FPG. Assessment based on bladder dysfunction severity (9 mild, 13 moderate, 8 severe cases) showed a progressive decline in VIP and 5-HT levels with increasing disease severity: the mild group had the highest values, followed by the moderate group, and the severe group showed the lowest levels. ANOVA revealed significant differences in VIP and 5-HT levels across severity groups (P<0.05). Post-hoc Tukey tests confirmed inter-group differences (P<0.05, Figure 2).

### Dynamic changes of VIP and 5-HT in DNB

**Figure 3:**
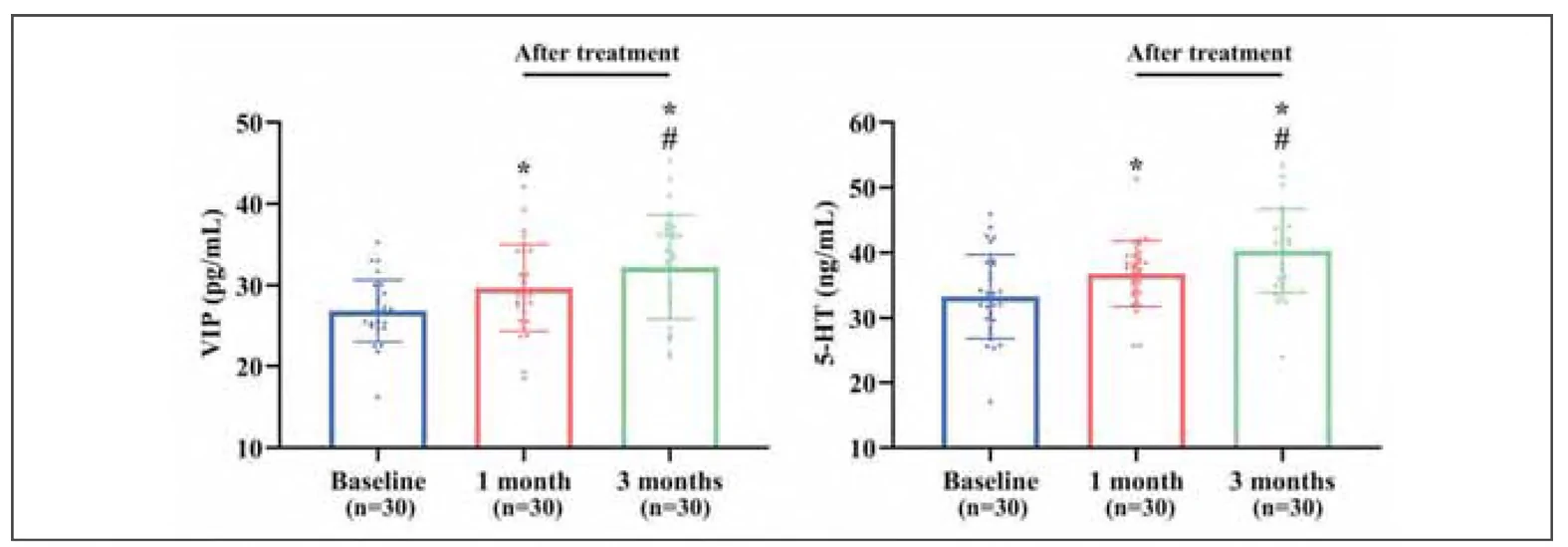
VIP, 5-HT changes before and after DNB treatment. * vs. 1 month after treatment P<0.05, # vs. 3 months after treatment P<0.05.

A significant increase in VIP and 5-HT from baseline was observed after one month of therapy (P<0.05), with levels demonstrating a further significant rise by the three-month mark (P<0.05). These findings indicate that the normalization of VIP and 5-HT is closely linked to the clinical management timeline of DNB (Figure 3).

### Link between VIP/5-HT and adverse bladder functional outcomes

In the course of follow-up, early signs of upper urinary tract injury were identified in 11 patients. While VIP and 5-HT concentrations showed no notable difference one month after treatment relative to those without injury (P>0.05), a significant reduction in both markers was detected at three months (P<0.05). Additionally, urinary tract infection was diagnosed in 8 patients. Consistent with the previous findings, these patients also demonstrated markedly lower VIP and 5-HT levels at the 3-month assessment compared to uninfected individuals (P<0.05, Table 2).

**Table 2:** Relationship between VIP5-HT and bladder function outcomes in groups with and without complications.

Projects	Time	Indicators	With complication<br>(n=19)	Without complication<br>(n=11)	t values	P values
Upper urinary <br>tract injury	1 month	VIP (pg/mL)	28.93±5.10	31.02±5.66	1.039	0.308
		5-HT (ng/mL)	36.32±5.78	37.67±3.43	0.701	0.489
	3 months	VIP (pg/mL)	34.94±5.88	27.64±4.31	3.584	0.001
		5-HT (ng/mL)	42.41±6.11	36.65±5.33	2.603	0.015
Projects	Time	Indicators	With complication <br>(n=22)	Without complication <br>(n=8)	t values	P values
Urinary tract<br>infections	1 month	VIP (pg/mL)	29.57±5.85	30.03±3.75	0.205	0.839
		5-HT (ng/mL)	37.28±4.84	35.53±5.63	0.839	0.409
	3 months	VIP (pg/mL)	34.57±5.24	25.93±4.88	4.065	<0.001
		5-HT (ng/mL)	42.22±5.58	35.01±5.71	3.114	0.004

### Predictive value of VIP and 5-HT for DNB disease progression

**Figure 4:**
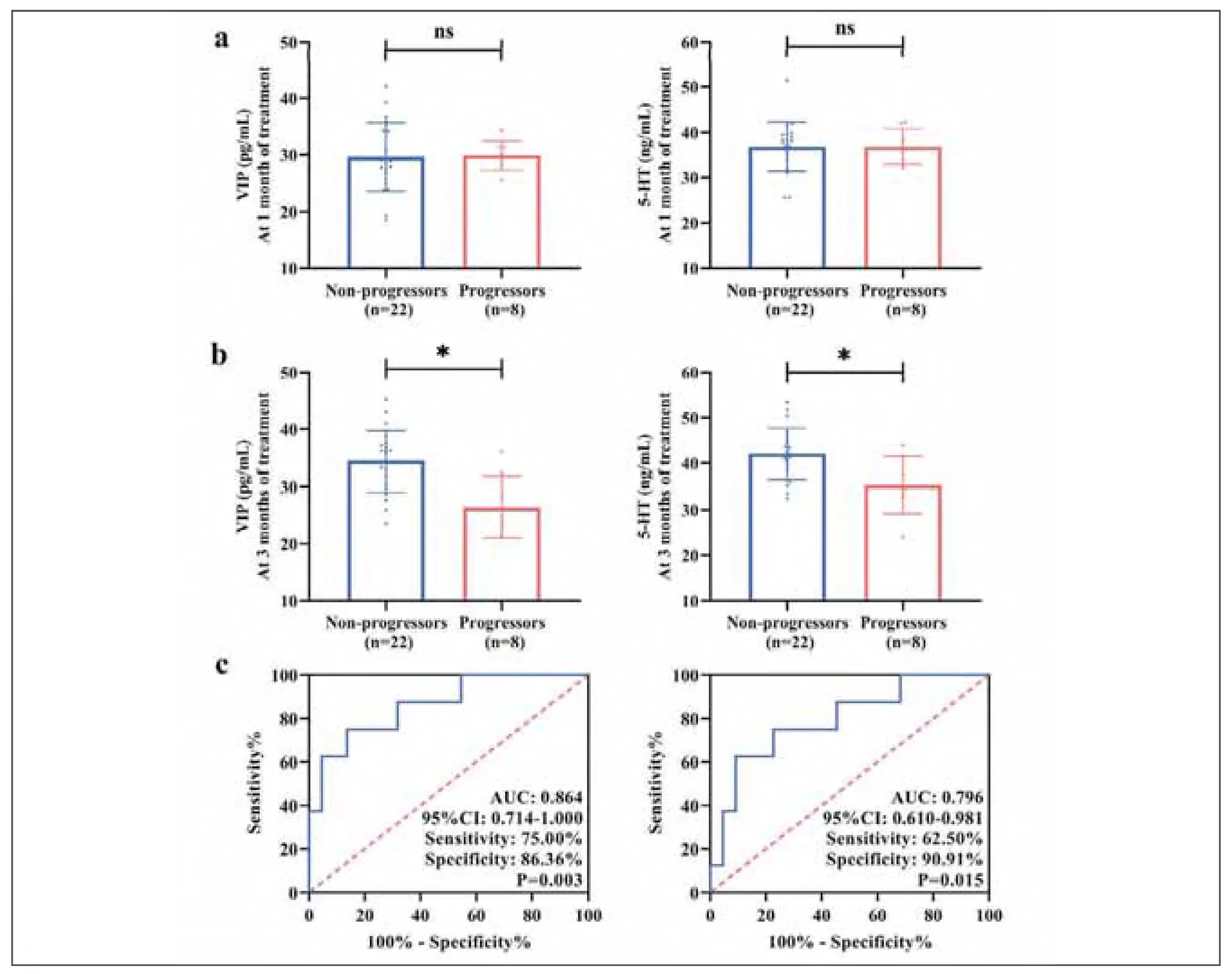
Effect of VIP,5-HT on the prediction of DNB disease progression. (a) Comparison of VIP,5-HT in patients with and without disease progression (1 month after treatment). (b) Comparison of VIP,5-HT in patients with and without disease progression (3 months after treatment). (c) Predictive value of VIP,5-HT for disease progression. *P<0.05, nsP>0.05.

Finally, evaluation using the mean monthly PVR growth rate identified eight subjects with disease progression. Patients who progressed exhibited lower VIP and 5-HT levels at 3 months post-treatment compared to non-progressors (P<0.05). ROC curve showed that the AUC of VIP and 5-HT in predicting the prognosis of DNB disease progression were 0.864 and 0.0.796, respectively (P<0.05), which had good reference value (Figure 4).

## Discussion

Through a combination of cross-sectional and longitudinal methodologies, this research systematically delineated the roles of VIP and 5-HT in DNB and their correlation with PVR. The findings are summarized as follows: ① Serum VIP and 5-HT were substantially lower in the DNB group relative to controls and varied inversely with PVR. ② The simultaneous assessment of both biomarkers surpassed the diagnostic performance of single-parameter detection. ③ Dynamic follow-up data showed that escalating VIP and 5-HT levels over the course of therapy were intimately linked to bladder functional recovery. ④ Assessment at three months revealed that diminished levels of VIP and 5-HT carried prognostic value, identifying patients at increased risk for upper urinary tract impairment, urinary tract infections, and clinical deterioration. The results suggest the involvement of VIP and 5-HT in DNB pathogenesis, possibly via their joint modulation of bladder neuromuscular function. Consequently, they are promising biomarkers for early detection, patient stratification, and prognostic assessment. However, this hypothesis requires validation through in vitro models examining neuronal-glial crosstalk or animal studies with knockout models.

VIP as a NANC neurotransmitter, has been proved by previous studies to participate in the regulation of urination by inhibiting detrusor overactivity and promoting bladder emptying [11]. Investigations in animal models reveal a direct association between suppressed VIP expression and diminished detrusor contractility [12]. This corroborates our observations that decreased VIP correlates with elevated PVR and marks disease advancement. However, existing research is predominantly confined to animal models or single population analyses [13][14], lacking investigation into dynamic VIP changes. This research, on the other hand, identified synchronized elevation in VIP expression with both treatment progression and urodynamic normalization, supporting its biomarker potential for therapeutic monitoring. 5-HT, a monoamine neurotransmitter, influences detrusor muscle contraction and afferent nerve signaling via its receptors [15]. Clinical studies have shown that there is a correlation between serum 5-HT level and bladder function [16]. However, its independent diagnostic capability continues to be contentious. Our cross-sectional analysis revealed an inverse correlation between 5-HT levels and PVR. Additionally, lower 5-HT was associated with higher UTI risk, potentially reflecting its role in maintaining bladder mucosal integrity [17]. It is important to highlight that earlier research largely focused on the isolated roles of VIP or 5-HT. In contrast, the present study provides the first evidence that simultaneous VIP and 5-HT measurement substantially improves diagnostic performance. This points to a synergistic relationship between VIP and 5-HT in DNB pathogenesis. The synergy between VIP and 5-HT may involve neuroendocrine axes: VIP modulates smooth muscle via cAMP-PKA signaling, while 5-HT regulates neuronal excitability through 5-HT2A/3A receptors. Their crosstalk in sacral micturition centers could amplify detrusor relaxation, as suggested by studies on autonomic neuropathy. Such an approach also introduces a promising method for early identification of DNB in individuals with asymptomatic or non-classical DM, potentially mitigating the risk of upper urinary tract impairment. The combined VIP and 5-HT detection outperformed traditional DNB markers such as nerve conduction velocity (AUC 0.70-0.75) [18], highlighting its superior diagnostic potential.

Preliminary data support the potential of VIP and 5-HT as biomarkers, but external validation is required before routine clinical application. A tiered »biomarker + imaging« assessment system should be established, combined with regular monitoring of PVR. At the same time, management should prioritize neurotransmitter-modulating therapies (e.g., 5-HT receptor agonists, VIP analogs) for patients with markedly low VIP and 5-HT, with ongoing assessment of treatment response. Finally, the integration of VIP, 5-HT, and the mean monthly growth rate of PVR into a DNB prognostic prediction model is paramount. This model will assist in risk stratification, enabling the implementation of reinforced follow-up plans for individuals at elevated risk.

Nevertheless, this research is subject to certain limitations. The longitudinal follow-up, while revealing VIP and 5-HT dynamics, lacked repeated measures across multiple timepoints, thereby undermining the robustness of the causal relationship. Although VIP and 5-HT show diagnostic promise, their routine screening requires cost-effectiveness analysis due to the higher expense of LC-MS compared to conventional tests. In addition, although the sample size estimation meets the statistical requirements (30 cases in each group), but due to the single-center recruitment strategy, which may introduce selection bias, the extrapolation of results still needs to be further validated. Furthermore, this study did not delve into the detailed regulatory mechanisms of VIP and 5-HT (e.g., neuroinflammation and oxidative stress). More comprehensive mechanistic insights could be gained through genomics or proteomics in future research. Finally, the possible influence of hypoglycemic agents and diuretics on neurotransmitter levels was not entirely excluded. Thus, more rigorous drug washout protocols are recommended in follow-up studies. Future multi-center studies with diverse cohorts are needed to validate these findings and enhance generalizability.

## Conclusion

This study reveals the synergistic regulation of VIP and 5-HT in DNB and its dynamic correlation with PVR. The results demonstrate that concurrently measuring VIP and 5-HT levels provides a robust tool for the early diagnosis, risk stratification, and prognosis assessment of DNB, thereby informing improved clinical strategies. Future work should expand cohorts, extend follow-up, and explore molecular mechanisms to translate these findings into clinical practice.

## Dodatak

### Funding

This study was supported by the Suqian 2023 Natural Science Fund Project (No.k202314).

### Availability of data and materials

The data that support the findings of this study are available from the corresponding author upon reasonable request.

### Conflict of interest statement

All the authors declare that they have no conflict of interest in this work.
